# Umbilical Granuloma: Frequency, Associated Factors, 10-Year Treatment Trends, and Effectiveness at a Single Hospital in Japan

**DOI:** 10.3390/jcm12186104

**Published:** 2023-09-21

**Authors:** Shigeo Iijima

**Affiliations:** Department of Regional Neonatal-Perinatal Medicine, Hamamatsu University School of Medicine, Hamamatsu 4313192, Japan; siijima@hama-med.ac.jp; Tel.: +81-53-435-2312

**Keywords:** umbilical granuloma, neonate, frequency, silver nitrate, betamethasone valerate, ethanol, ligation

## Abstract

Umbilical granuloma (UG) is a common problem during the neonatal period; however, its epidemiology and etiology are poorly studied, and the best treatment option has not yet been established. We examined the medical records of neonates who were born and underwent 1-month evaluations at our hospital between 2013 and 2022 to investigate the frequency of—and factors associated with—UG, as well as the annual trends of UG treatments and their efficacy. Of the 6680 eligible neonates, 395 (5.9%) had UG. The annual incidence rate ranged from 3.8% to 7.3%. Gestational age, birth weight, and incidence of meconium-stained amniotic fluid were significantly associated with UG. Silver nitrate cauterization was the predominant UG treatment from 2013 to 2016. Silver nitrate cauterization and topical betamethasone valerate were nearly equally applied in 2017. Betamethasone application became predominant in 2018. The healing rates during the initial treatment period were 91% for silver nitrate cauterization, 97.7% for betamethasone application, 60% for ethanol disinfection, and 88% for ligation; these rates were significantly different (*p* < 0.001). Topical steroid application may be the most effective treatment. If steroid application is ineffective, then silver nitrate cauterization and ligation may be important treatment options.

## 1. Introduction

Umbilical granuloma (UG) is a common benign umbilical abnormality experienced by neonates. It is usually defined as a mass of moist, fleshy, pale-red granulation tissue at the base of the umbilicus after umbilical cord detachment. The tissue is soft, 3 to 10 mm in size, vascular, granular, and dull red or pink; furthermore, seropurulent discharge may occur [1]. It has been suggested that inflammatory processes are involved in granuloma formation. The normal umbilical cord usually detaches within 7 to 10 days after birth. Approximately 1 or 2 weeks after the cord separates, the remaining granulation tissue typically disappears with epithelization of the umbilical cord [2]; however, if the umbilical cord is not sufficiently epithelialized, then the granulation tissue becomes granuloma [1]. The mode of delivery, umbilical care method, moisture in the umbilicus, infection, and foreign objects are thought to be associated with UG [3,4,5]. In addition, UG has been associated with delayed cord separation, which is known to be associated with bacterial infection or some immune disorders (e.g., leukocyte adhesion defects, neutrophil dysfunction) [6]. However, despite several investigations, its etiology is not yet fully known.

UG can be treated surgically or non-surgically [7]. In addition to surgical therapy, silver nitrate cauterization is the most commonly used treatment modality [8,9]. Similar to other expectant management methods, the various options that have been attempted include the topical application of copper sulfate, steroids, antibiotics, table salt, ethanol wipes, cryocautery, and electrocautery [8]. However, there is no clear consensus regarding the optimal treatment modality for UG.

The present study aimed to determine the frequency of, and factors associated with, UG experienced by neonates, investigate the historical trends of UG treatment at a single institution in Japan, and evaluate the efficacy of different therapeutic approaches for UG.

## 2. Materials and Methods

### 2.1. Patients and Data Collection

We conducted a retrospective chart review of consecutive healthy neonates who were born at Hamamatsu University Hospital between 1 January 2013 and 31 December 2022, and underwent 1-month evaluations; traditionally, in Japan, all neonates undergo a 1-month evaluation, usually at the hospital or clinic where they were born. To assess the development of UG among healthy neonates, we excluded neonates with low birthweight (<2300 g), those who were delivered preterm (<36 weeks of gestation), those with congenital anomalies and/or chromosomal abnormalities, and those admitted to the neonatal intensive care unit.

Eligible neonates were divided into two groups according to the presence or absence of UG. Statistical analyses were performed to investigate the incidence of and etiological factors associated with UG. Among neonates with UG, the treatment modalities were statistically analyzed to evaluate their efficacy.

Data regarding sex, birth weight, gestational age, delivery mode, presence of meconium-stained amniotic fluid (MSAF), intrauterine infection, 1-minute Apgar score, and umbilical arterial blood pH were obtained as potential predictors of UG formation. Additionally, the examination records of the 1-month evaluation were obtained. The presence of UGs, treatment of UGs, and efficacy of treatment were reviewed using these examination records.

### 2.2. Ethical Approval

The present study was designed and conducted in line with the ethical principles of the Declaration of Helsinki, and it was approved by the ethics committee of Hamamatsu University School of Medicine (approval number: 22–173). During this retrospective study, the parents of the patients were not required to provide informed consent because the analysis used anonymous clinical data that had been obtained after each patient’s parents provided written consent for clinical management. Moreover, an optional method of providing consent was available via the hospital’s website.

### 2.3. Collection of Seasonal and Meteorological Data

Infection is one of the causes of UG [1] and is known to be associated with high temperatures and humidity. Considering global warming, during the present study, we decided to investigate the relationship between the incidence of UG and season and/or weather (temperature and humidity). Japan is an island country with four distinct seasons comprising: cold winters, hot and humid summers, and two intermediate phases (spring and autumn) characterized by mild weather. Hamamatsu City is located in the central region (latitude, 34.7°N) and has an annual average temperature of 16.6 °C and annual average relative humidity of 67%. During this study, the effects of temperature and humidity on UG development were examined separately during seasons with average temperatures higher than 20 °C and average relative humidity higher than 70% (May to October), as well as during other seasons (November to April), based on the data of monthly weather during 1991 to 2020 that were recorded by the Hamamatsu Local Meteorological Observatory and published on the Japan Meteorological Agency website [10].

### 2.4. Routine Umbilical Care and Treatment Strategies for Umbilical Granuloma

It was previously reported that umbilical cord clamp position was associated with the incidence of UGs [5]. At our hospital, after birth, the umbilical cord of the neonate is clamped, cut at a distance of 2 to 3 cm from the umbilical base (usual method performed in Japan), and cleaned once daily with ethanol wipes, dressings are not applied. The mothers of the neonates are instructed by the midwives to continue umbilical cord care at home until the 1-month evaluation. These umbilical cord care methods have remained unchanged during the 10-year study period.

UGs were diagnosed by an experienced pediatrician after performing a thorough examination to exclude other common umbilical problems. In particular, umbilical polyps, which represent a distal omphalomesenteric remnant, appear similar to UGs. The differences shown in Table 1 are used as references [11,12].

UGs have been treated using the following four modalities: chemical cauterization with silver nitrate; topical application of steroid ointment; ethanol disinfection; and ligation. The pediatrician in charge of the patient chose the modality based on the condition of the UG.

#### 2.4.1. Silver Nitrate Cauterization

Silver nitrate cauterization was performed only once by a pediatrician during the 1-month evaluation. The UG was cauterized with 20% silver nitrate solution until it turned gray–white. The silver nitrate solution was applied using a cotton swab. After cauterization, the excess silver nitrate was thoroughly neutralized with saline solution, and the excess liquid was removed using a gauze wipe. The parents of the patients were informed about the possible side effects, including chemical burns of the periumbilical area and pigmentation changes in the cauterized area.

#### 2.4.2. Topical Steroid Application

A 0.12% betamethasone valerate ointment, which is a Japanese class III (strong) steroid, was applied to the lesion twice daily at home. An adequate amount of the ointment was applied to the entire UG surface. No culture tests were performed and no antimicrobial treatments were administered during this time. Steroid ointment application at home was performed for a maximum of 2 weeks. The patients’ parents were informed about the possible side effects, including local infection, pigmentation, and skin color changes.

#### 2.4.3. Ethanol Disinfectant

The UG was cleaned with ethanol wipes once daily after bathing at home.

#### 2.4.4. Ligation

Ligation was performed by a pediatrician during the 1-month evaluation. The UG was ligated at its base using 3-0 silk sutures.

#### 2.4.5. Treatment Outcomes

If the aforementioned treatments were effective, then the discharge volume decreased and eventually disappeared. The UG gradually shrank, darkened, turned black, dried, and subsequently separated. No response was defined as persistent umbilical discharge or no regression of the UG. After the pediatrician treated the UG during the 1-month evaluation, the parents were told that if the UG did not heal within 2 weeks, or if any symptoms of suspected side effects appeared, then they should bring the patient back to the hospital for additional evaluation. During the additional evaluation, the physician determined the efficacy of the initial treatment. If the treatment was ineffective, then another treatment method was applied. Lesions that did not respond to two courses of treatment were surgically resected.

### 2.5. Statistical Analysis

The data obtained during this study were analyzed using the Statistical Package for Social Sciences (SPSS version 25; IBM Corporation, Armonk, NY, USA). Data are presented as medians with interquartile ranges for continuous variables and are presented as numbers and percentages for categorical variables. Non-parametric methods (Spearman’s correlation coefficient, Kruskal–Wallis test, and Mann–Whitney U test) were used to assess the influence of clinical and seasonal parameters on UG development and evaluate the efficacy of UG treatments. A linear regression model was constructed using UG development and the aforementioned parameters as independent and dependent variables. All statistical tests were two-sided, and *p* < 0.05 was considered statistically significant.

## 3. Results

### 3.1. Samples

Of the 6680 eligible neonates, 3349 (50.1%) were male. The median gestational age and birth weight were 39.14 weeks and 2989 g, respectively. Additionally, 4418 (66.1%) neonates were born via vaginal delivery, 586 (8.8%) were born via vacuum extraction, and 1663 (24.9%) were born via cesarean delivery.

### 3.2. Incidence of Umbilical Granuloma and Associated Factors

UGs were detected in 395 patients. The annual incidence rates of UG vary, with a minimum of 3.8%, maximum of 7.3%, and median of 6.3% observed (Table 2). According to the trends observed over the 10-year study period, this rate has increased; moreover, this rate particularly increased after 2016. The results of a comparison of the demographic, clinical, and seasonal characteristics of the neonates with and without UGs are shown in Table 3. UG was significantly more common among male neonates than among female neonates (*p* = 0.049). The median gestational age and birth weight were 39.43 weeks and 3066 g, respectively, for neonates with UG; however, they were 39.14 weeks and 2984 g, respectively, for neonates without UG. There were significant differences in gestational age (*p* < 0.001) and birth weight (*p* < 0.001) associated with the occurrence of UG. Regarding the delivery mode, 270 (68.4%) neonates with UG were born via vaginal delivery, 47 (11.9%) were born via vacuum extraction, and 75 (19.0%) were born via cesarean delivery; in contrast, 4148 (66.0%) neonates without UG were born via vaginal delivery, 539 (8.6%) were born via vacuum extraction, and 1588 (25.3%) were born via cesarean delivery. There was no statistically significant relationship between UG development and vaginal delivery; however, UGs were significantly more common among neonates born via vacuum extraction (*p* = 0.024) and less common among those born via cesarean delivery (*p* = 0.005). The incidence of MSAF was significantly higher among neonates with UG than among those without UG (20.8% vs. 14.3%; *p* < 0.001). No statistically significant relationships between UG development and the incidence of intrauterine infection, pH of blood obtained from the umbilical artery, and the 1-minute Apgar score were observed. There was no statistically significant relationship between UG development and seasonal changes in temperature or humidity.

### 3.3. Umbilical Granuloma Treatment and Its Effectiveness

**Figure 1:**
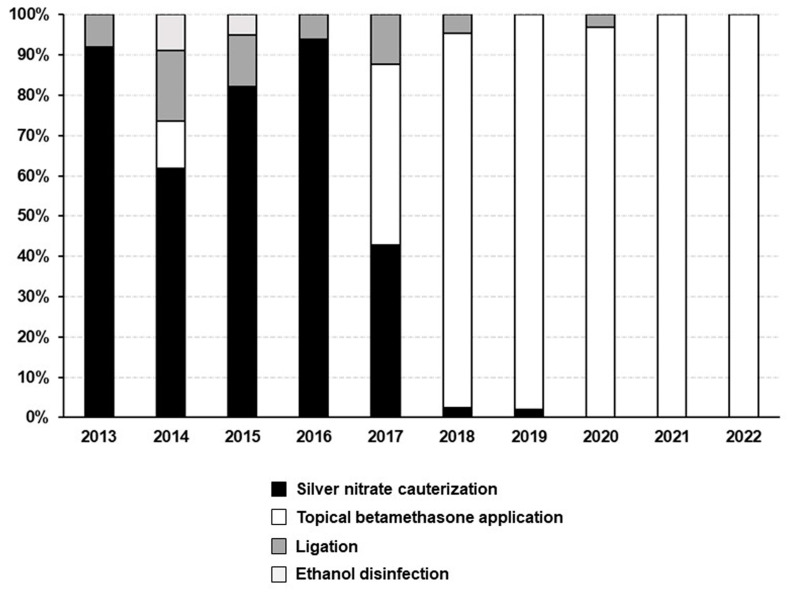
Annual trends of treatments for umbilical granuloma from 2013 to 2022.

During the 10-year study period, 395 neonates received treatment for UG: silver nitrate cauterization was performed for 145 cases (36.7%); topical application of betamethasone valerate ointment was performed for 220 cases (55.7%); ligation was performed for 25 cases (6.3%); ethanol disinfection was performed for five cases (1.3%). The annual trends of the treatment methods are shown in Figure 1. Silver nitrate cauterization was the predominant method used between 2013 and 2016. However, in 2017, silver nitrate cauterization and topical application of betamethasone were used almost equally. In 2018, betamethasone application became the predominant treatment.

**Figure 2:**
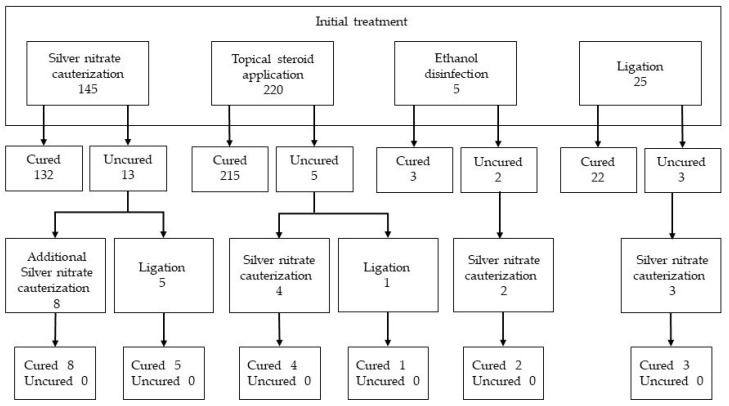
Flow diagram of the initial and additional treatment modalities and their efficacy.

There were no significant differences in the characteristics of the neonates administered the four treatments (Table 4). Figure 2 shows the flow diagram of the initial and additional treatment modalities and their efficacy. During the 2-week observational period, 23 patients presented to the pediatric outpatient clinic of our hospital. Thirteen UG cases treated with silver nitrate cauterization as the initial therapy failed to respond to treatment; therefore, eight of these cases required additional silver nitrate cauterization, and five required ligation. Five of the cases treated with topical betamethasone showed no response to treatment; therefore, four cases underwent silver nitrate cauterization and one case required ligation. Three cases that underwent ligation did not achieve a cure and required silver nitrate cauterization. Two cases treated with ethanol disinfection showed no response; therefore, silver nitrate cauterization was performed. During the following 2-week observational period, the patients did not experience failure of the UG to heal; therefore, they did not return to the hospital. Furthermore, the patients did not return to the hospital because they did not require treatment for side effects during the entire observational period.

A comparison of the UG healing rates after the initial treatment showed a significant difference among the four treatments (*p* < 0.001). The healing rates were as follows: 91% for silver nitrate cauterization; 97.7% for betamethasone application; 60% for ethanol disinfection; and 88% for ligation. A comparison of silver nitrate cauterization and betamethasone application also showed a statistically significant difference (*p* = 0.002).

## 4. Discussion

According to the results of the present study, the annual incidence rate of UG among neonates ranged from 3.8% to 7.3%. Few studies have investigated the frequency of UG. To the best of our knowledge, this is the first report of UG incidence in Japan. Assi et al. reported a prevalence of 0.2% (1 in 500 neonates) [13], which has been cited by many studies. In contrast, Tülin et al. studied more than 20,000 neonates and reported a UG incidence of 3.83% [3]. Other studies have reported UG frequency rates as high as 8.6% to 12.8% [14,15,16] and as low as 1% to 2% [17,18]. During the present study, the incidence of UG showed an increasing trend over the course of the 10-year study period. Furthermore, during the study period, umbilical cord care, the definition of UG, and physicians’ diagnostic skills did not change. Additionally, there was no apparent change in the severity of UG. Considering the increased incidence that occurred after 2016, additional comparisons of demographic, clinical, and seasonal/meteorological factors observed from 2013 to 2015 and from 2016 to 2022 were conducted; however, relevant factors were not detected.

The multiple linear regression analysis revealed that male sex, longer gestational period, and higher frequency of MSAF were associated with UG development. The frequency of UG is the same among male and female neonates [4,5]; however, one study reported that the male-to-female ratio was 1.1:1 [19], whereas another reported that the male-to-female ratio was 1.7:1 [4]. During our study, the UG frequency was significantly higher among male neonates, with a male-to-female ratio of 1.2:1. UG develops because of mild inflammation of the non-epithelialized granulation tissue in the umbilicus [4,17,20]. Inflammatory responses are sex-dependent. In vivo studies have demonstrated a greater release of proinflammatory cytokines after in vivo lipopolysaccharide stimulation in male fetuses than in female fetuses [21,22]. During our study, the UG incidence was associated with gestational age and MSAF; however, there was no association with fetal distress (umbilical cord pH or 1-minute Apgar score) or intrauterine infection, which are factors associated with MSAF. The frequency of MSAF increases with gestational age [23,24]. MSAF is more likely to cause intrauterine inflammation than clear amniotic fluid [21]. Therefore, as the fetus matures, the amniotic fluid becomes more turbid, and inflammation of the umbilical cord may continue after birth, leading to the development of UG. High temperatures and humidity increase bacterial growth, which may lead to the development of UG. In Japan, the typical inland climate comprises large variations in temperature and humidity between summer and winter. Therefore, we examined seasonal variations in the UG incidence. This assessment has not been performed during other studies; however, the results showed no significant seasonal variations.

Silver nitrate cauterization is the most commonly used treatment for UG [1]. Silver nitrate causes strong protein coagulation and has antiseptic, astringent, and bactericidal effects; furthermore, it is used to remove granulation because it destroys and removes inflamed tissue [25]. In Japan, the production and sales of silver nitrate sticks were discontinued in 1997 because of unprofitability; however, many institutions continue to use silver nitrate sticks. The supply of silver nitrate sticks at our clinic was depleted in 2012; therefore, we have been using a 20% silver nitrate solution that was adjusted to create an in-house preparation. Silver nitrate is classified as a pharmaceutical product; however, case reports of burning of the surrounding skin after its application have been published [26,27,28,29]. Silver nitrate is a strong caustic chemical compound that has the ability to corrode or burn skin tissues on contact, primarily because of its high reactivity with proteins and other organic molecules found in skin cells. Burns caused by silver nitrate have been reported with the use of both 75% silver nitrate sticks and 20% silver nitrate solution [28,29].

Recently, steroid application has been reported to be effective for the treatment of UG. In 2015, Brødsgaard et al. compared the therapeutic efficacy of silver nitrate cauterization and 0.06% clobetasol propionate application (twice daily) for 109 neonates with UGs and reported that the healing rates of the silver nitrate and clobetasol groups at 30 days were similar (silver nitrate 96% vs. clobetasol 90%) [30]. After it was reported that steroid application was effective for UG, treatment for UG at our hospital shifted from silver-nitrate-based treatment to steroid-application-based treatment after 2017. In 2018, Ogawa et al. studied the efficacy of the topical application of 0.12% betamethasone valerate ointment (twice daily) and compared it to that of silver nitrate (20%) cauterization (once weekly) among 207 neonates with UGs during a multicenter, randomized, controlled trial [31]. The results showed that the healing rates of the betamethasone and silver nitrate groups were 87.5% and 82.0%, respectively, on day 14, and 90.4% and 91.0%, respectively, on day 21, indicating almost equal treatment efficacy. Corticosteroids have potent anti-inflammatory and immunosuppressive effects, and topical steroids exert their anti-inflammatory effects by suppressing inflammatory cell function and locally inducing cell death in the skin [32]. Furthermore, recent studies have found that the anti-inflammatory effects of topical steroids may contribute to a decreased number of fibroblasts [33]. Therefore, topical steroid application is believed to exert a therapeutic effect by inhibiting the progression of the inflammatory process leading to the proliferation of fibroblasts, which are the main components of UG development. However, appropriate administration techniques are required to obtain the desired therapeutic results. Moreover, in cases of high-dose and/or long-term use, it is necessary to consider the risks of local skin atrophy and hypopigmentation, capillary vasodilation, bacterial infection caused by local immunosuppressive effects, and systemic side effects such as suppression of the hypothalamic–pituitary–adrenal axis [34,35]. However, during previous studies, the adverse effects of topical steroid administration were mild and reversible [30,31]. During our study, a small amount (<5 g) of betamethasone valerate was administered to each neonate during the entire investigation period (<2 consecutive weeks), with no local or systemic side effects. Therefore, topical betamethasone application can be considered an alternative to silver nitrate cauterization, with the same or superior effectiveness; furthermore, it can be administered by parents at home, with minimal adverse effects.

Topical disinfection is expected to reduce bacterial growth at the umbilical base [36]. Daniels et al. demonstrated that two-thirds of all UGs resolve within 3 weeks when parents clean them with ethanol wipes during each diaper change [26]. However, there is no evidence of its effects on UG. Brødsgaard et al. compared the efficacy of silver nitrate cauterization, steroid application, and ethanol disinfection for the treatment of UG and found that the average time required for healing was 12.9 days for silver nitrate, 17.4 days for steroids, and 27 days for disinfection. The healing rate after 30 days was more than 90% for both silver nitrate and steroids but it was 53% for disinfection, which was clearly inferior [30]. During the present study, ethanol disinfection resulted in the lowest healing rate; however, the number of patients treated with disinfectant was too small (five cases) to draw conclusions about the effectiveness of this treatment.

To date, no study has compared the efficacy of ligation with that of other treatment options. Our study showed that ligation was less effective than silver nitrate cauterization and betamethasone application. Because most UGs are located relatively deep in the umbilical region and are difficult to locate, ligating the granuloma at its base is technically difficult. Additionally, the complications of occasional bleeding during ligation and bleeding caused by granuloma dissection during ligation are possible [8]. Ligation is not always an appropriate alternative but it can be useful for specific UG types, such as those that are pedunculated.

All 23 cases involving failure of the initial treatment were cured by silver nitrate cauterization or ligation as an additional treatment. Silver nitrate cauterization and ligation are effective treatment options despite the severity of their side effects and procedure difficulty. Therefore, we believe that these treatments should be administered as additional treatments when steroid use is ineffective.

During the present study, the healing rates of silver nitrate cauterization, steroid application, and ligation were high; however, to evaluate the effectiveness of these treatments, the advantages and disadvantages as well as the healing rate of each treatment modality must be considered.

Our study included a relatively large number of neonates who were born at a single hospital and received the same umbilical cord care. We examined the 10-year trends of UG treatment, observed a marked change in first-line treatment, and analyzed the background of this change. The results of this study provide valuable insights for national and global medical communities.

One limitation of our study was that it was conducted retrospectively. Moreover, over the course of the 10-year study period, different patients were treated with different treatment methods, with varying rates of use for each method; therefore, an accurate assessment of the effectiveness of each treatment method was difficult. Furthermore, the treating physician instructed the parents of the neonates with UG to revisit our hospital only when treatment was ineffective. The physician did not directly determine the effectiveness of the treatment by instructing the parents to bring the patients to the hospital on a regular basis for evaluation. Moreover, the parents of the patients were instructed to return to the hospital if the patients experienced treatment side effects; however, in the case of minor side effects, parents may not have been aware of the symptoms, or they may have considered returning to the hospital to be unnecessary. This may have created the risk of bias in the determination of the efficacy and safety of treatment. Well-designed, large-scale, prospective studies are required to address these issues.

Recent studies performed in developing countries have shown the therapeutic efficacy and safety of salt application for UG [3,4]. Additionally, Janyoungsak et al. compared the efficacy of 3% NaCl solution and that of 30% NaCl solution for treating UG and showed the efficacy of hypertonic NaCl solution [37]. However, there is no record of the use of these treatments in Japan. Therefore, a comparison of these treatments and steroid administration should be conducted in the future.

## 5. Conclusions

The incidence rate of UG at a tertiary hospital in Japan ranged from 3.8% to 7.3%. UG was more common among male neonates and associated with longer gestational periods and MSAF, suggesting that UG development is caused by an inflammatory process. The UG healing rates attributable to topical steroid application, silver nitrate cauterization, and ligation were high. However, considering the healing rate and advantages and disadvantages of each treatment modality, steroid application might be the best initial treatment. Silver nitrate cauterization and ligation might be useful additional treatments when topical steroid ointment application is ineffective. Pediatricians should consult a surgeon when lesions do not respond to two courses of treatment.

## Figures and Tables

**Table 1:** Differences between umbilical granulomas and umbilical polyps.

	Umbilical Granuloma	Umbilical Polyp
Onset	A few weeks after birth	After umbilical cord separation
Color	Dull red or pink	Cherry red
Surface characteristics	Unevenness	Smooth
Hardness	Soft	Hard
Secretions	Serous bloody, sometimes purulent	Serous
Pathology	Granulation tissue	Smooth muscle tissue
Non-surgical treatment	Effective	Not effective
Ultrasonography findings	Hypervascular	Hypovascular

**Table 2:** Annual incidence rates of umbilical granuloma between 2013 and 2022.

Year	Eligible Neonates (No.)	Neonates with Umbilical Granuloma (No.)	Annual Incidence Rate of Umbilical Granuloma (%)
2013	659	25	3.8
2014	697	34	4.9
2015	794	39	4.9
2016	784	49	6.3
2017	711	49	6.9
2018	686	43	6.3
2019	682	50	7.3
2020	628	33	5.3
2021	543	38	7.0
2022	496	35	7.1
Total	6680	395	5.9

**Table 3:** Demographic and clinical characteristics of neonates with or without umbilical granuloma.

	Neonates with UG	Neonates without UG	Simple Linear Regression *p*-Value	Multiple Linear Regression *p*-Value
	*n* = 395	*n* = 6285		
Gestational age, weeks	39.43 (38.57–40.28)	39.14 (38.14–40.0)	<0.001	<0.001
Birth weight, g	3066 (2802–3282)	2984 (2740–3236)	0.001	NS
Male sex, *n* (%)	217 (54.9)	3132 (49.8)	0.049	0.030
Delivery mode				
Spontaneous vaginal delivery, *n* (%)	270 (68.4)	4148 (66.0)	0.334	NS
Vacuum extraction, *n* (%)	47 (11.9)	539 (8.6)	0.024	NS
Cesarean delivery, *n* (%)	75 (19.0)	1588 (25.3)	0.005	NS
Meconium-stained amniotic fluid, *n* (%)	82 (20.8)	896 (14.3)	<0.001	0.032
Intrauterine infection, *n* (%)	1 (0.25)	25 (0.40)	0.654	NS
One-minute Apgar score	8 (8–9)	8 (8–9)	0.801	NS
Umbilical arterial blood pH	7.316 (7.274–7.354)	7.312 (7.276–7.348)	0.317	NS
Birth during a warm and humid season *, *n* (%)	218 (55.2)	3329 (53.0)	0.339	NS

Categorical variables are expressed as numbers (%). Continuous variables are expressed as medians (interquartile ranges). * A warm and humid season refers to a season with an average temperature higher than 20 °C and average relative humidity higher than 70% (May to October). UG, umbilical granuloma; NS, not significant.

**Table 4:** Demographic, clinical, and seasonal characteristics of 395 neonates who received one of four treatments for umbilical granuloma.

	Treatment				*p*-Value
	Silver Nitrate Cauterization	Betamethasone Valerate Application	Ethanol Disinfection	Ligation	
	*n* = 145	*n* = 220	*n* = 5	*n* = 25	
Male sex, *n* (%)	77 (53.1)	121 (55.0)	2 (40.0)	17 (68.0)	0.500
Gestational age, weeks	39.57	39.28	39.14	39.71	0.228
	(38.57–40.43)	(38.57–40.28)	(38.36–40.42)	(39.14–40.71)	
Birthweight, g	3036	3072	3140	3068	0.861
	(2768–3288)	(2833–3293)	(2500–3274)	(2799–3182)	
Delivery mode					
Spontaneous vaginal delivery, *n* (%)	97 (66.9)	149 (67.7)	4 (80.0)	20 (80.0)	0.560
Vacuum extraction, *n* (%)	22 (15.2)	22 (10.0)	1 (20.0)	2 (8.0)	0.406
Cesarean delivery, *n* (%)	26 (17.9)	46 (20.9)	0 (0.0)	3 (12.0)	0.459
Meconium-stained amniotic fluid, *n* (%)	25 (17.2)	48 (21.8)	3 (60.0)	6 (24.0)	0.108
Intrauterine infection, *n* (%)	0 (0)	1(0.5)	0(0)	0 (0)	0.851
One-minute Apgar score	8 (8–9)	8 (8–8)	9 (8–9)	8 (8–9)	0.032
Umbilical arterial blood pH	7.314	7.316	7.371	7.323	0.656
	(7.268–7.353)	(7.278–7.353)	(7.128–7.394)	(7.289–7.360)	
Birth during a warm and humid season *, *n* (%)	70 (48.3)	133 (60.5)	2 (40.0)	14 (56.0)	0.126

Categorical variables are expressed as numbers (%). Continuous variables are expressed as medians (interquartile ranges). * A warm and humid season refers to a season with an average temperature higher than 20 °C and average relative humidity higher than 70% (May to October).

## Data Availability

The datasets supporting the conclusions of this study are included in this article.
